# Long-term and short-term preservation strategies for tissue engineering and regenerative medicine products: state of the art and emerging trends

**DOI:** 10.1093/pnasnexus/pgac212

**Published:** 2022-09-30

**Authors:** Sara Freitas-Ribeiro, Rui L Reis, Rogério P Pirraco

**Affiliations:** 3B’s Research Group, I3Bs—Research Institute on Biomaterials, Biodegradables and Biomimetics, University of Minho, Headquarters of the European Institute of Excellence on Tissue Engineering and Regenerative Medicine, AvePark, Parque de Ciência e Tecnologia, Zona Industrial da Gandra, 4805-017 Barco GMR, Portugal; ICVS/3B’s—PT Government Associate Laboratory, 4805-017 Barco GMR, Portugal; 3B’s Research Group, I3Bs—Research Institute on Biomaterials, Biodegradables and Biomimetics, University of Minho, Headquarters of the European Institute of Excellence on Tissue Engineering and Regenerative Medicine, AvePark, Parque de Ciência e Tecnologia, Zona Industrial da Gandra, 4805-017 Barco GMR, Portugal; ICVS/3B’s—PT Government Associate Laboratory, 4805-017 Barco GMR, Portugal; 3B’s Research Group, I3Bs—Research Institute on Biomaterials, Biodegradables and Biomimetics, University of Minho, Headquarters of the European Institute of Excellence on Tissue Engineering and Regenerative Medicine, AvePark, Parque de Ciência e Tecnologia, Zona Industrial da Gandra, 4805-017 Barco GMR, Portugal; ICVS/3B’s—PT Government Associate Laboratory, 4805-017 Barco GMR, Portugal

**Keywords:** slow freezing, vitrification, dry state preservation, hypothermic preservation, normothermic preservation

## Abstract

There is an ever-growing need of human tissues and organs for transplantation. However, the availability of such tissues and organs is insufficient by a large margin, which is a huge medical and societal problem. Tissue engineering and regenerative medicine (TERM) represent potential solutions to this issue and have therefore been attracting increased interest from researchers and clinicians alike. But the successful large-scale clinical deployment of TERM products critically depends on the development of efficient preservation methodologies. The existing preservation approaches such as slow freezing, vitrification, dry state preservation, and hypothermic and normothermic storage all have issues that somehow limit the biomedical applications of TERM products. In this review, the principles and application of these approaches will be summarized, highlighting their advantages and limitations in the context of TERM products preservation.

## Introduction

**Fig. 1.**
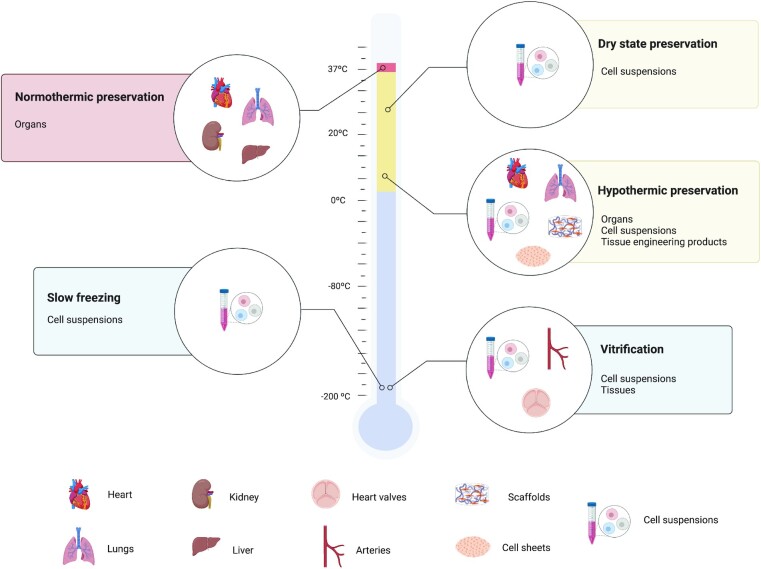
Overview of existing preservation strategies and respective applications. The preservation of TERM products, cells, tissues, and organs can involve different preservation strategies such as slow freezing, vitrification, dry state preservation, hypothermic and normothermic preservation. Preservation techniques are represented by their working temperature (Normothermic: 37°C, Dry state preservation: ≈20°C, Hypothermic preservation: 4°C, Slow freezing: -196°C, Vitrification: -196°C). Created with BioRender.com.

Finding solutions to address tissue loss and organ failure is a daily challenge for clinicians, who conventionally use tissues and organs sourced from suitable donors. In this case, however, the ever-growing demand already vastly exceeds the supply (1). The increasing prevalence of chronic diseases such as diabetes, cardiovascular disorders, obesity, as well as other severe medical conditions, are among the key factors driving this growth, that, in turn, represents an increase in healthcare costs. Heart transplantation procedures represented an expense of approximately 134 million euros in 2019 (2), while donation and transplantation of blood, tissues, and cells are estimated to represent around 6 billion euros per year in the EU alone (3). Tissue engineering and regenerative medicine (TERM) represent potential solutions to this problem and have therefore been attracting increased interest from clinicians (4). Tissue engineering approaches, in particular, typically use bioartificial 3D matrices termed scaffolds, composed of the most diverse materials, where cells are seeded and allowed to grow, to yield the desired tissue-like constructs. Tissue-like constructs are also possible to be fabricated in a scaffold-free manner, where only cells and their ECM comprise the construct (5–8). In fact, the use of such cell rich constructs has long been applied for the treatment of severe burns (9, 10) and corneal regeneration (11) in humans, demonstrating the efficacy of these strategies. The process of developing such potentially lifesaving constructs requires long periods of time since it involves the isolation and expansion of cells, construct fabrication, and maturation in specialized facilities before it is ready for application in the patient. The transportation step from the specialized facilities to the bedside is of great importance since all the desired properties of the constructs—largely dependent on cell viability—must be preserved up until implantation in a patient. Failure to adequately address this preservation issue gravely jeopardizes TERM constructs quality and therefore their efficacy and safety in patients. Recent articles addressing the problematics of shipping and logistics for TERM products are unanimous in recognizing preservation methodologies for storage and transportation as a key challenge to be surpassed for the widespread clinical application of TERM solutions (12–14). Furthermore, the development of advanced storage protocols for TERM products could also facilitate the development of “off-the-shelf” solutions, obviating patient waiting times and eliminating logistical concerns related with transporting the products through long distances. This would also have dramatic effects in the costs of the engineered products, which would further facilitate their clinical adoption. Table S1 summarizes some of the of studies reporting strategies for the preservation of tissue engineering products. Among the existing preservation strategies, long-term preservation protocols are effective for single cell preservation, but present added difficulties in fulfilling the necessary requirements for the successful preservation of 3D structures. Normo- or hypothermic protocols have already proven successes in preserving tridimensional biologic structures such as organs and thus their application in TERM products could be key to streamline their clinical translation. Therefore, the aim of this review is to provide an overview of the existing preservation strategies, slow freezing, vitrification, dry state preservation, hypothermic preservation, and normothermic preservation, (Fig. 1) focusing on the increasing evidence of the advantage of the use of hypothermic and normothermic temperatures as short-term preservation strategies for TERM products.

## Long-term preservation

### Cryogenic preservation

Cryogenic preservation, or cryopreservation, represents one of the oldest and the most common storage processes. It consists of using very low temperatures to preserve living cells and tissues for long periods of time. Depending on cell type or tissue, there is a great diversity of cryobiological responses during the freezing and thawing cycle. Considering this, several cryogenic processes have been developed, namely slow freezing and vitrification. While different, all these processes involve the common steps of mixing a cryoprotectant with cells or tissues, cooling to a low temperature and finally removing the cryoprotectant from cells or tissues after thawing. The use of cryoprotectants is, in fact, key to a successful cryogenic preservation since the viability of the preserved sample will largely depend on the choice of an adequate cryoprotectant (15). Their role is to stabilize the cell membrane, minimize osmotic stress, and protect cells from intra and extracellular ice formation (16) by reducing the water content in cells/tissues and increasing the total concentration of all solutes. There are several types of cryoprotectants, ranging from low molecular weight solutes (permeating) to sugars and high molecular weight polymers (nonpermeating). The effectiveness of a given cryoprotectant usually depends on its permeability and toxicity to cells. Permeating cryoprotectants, such as glycerol and dimethyl sulfoxide (DMSO), can penetrate the cell membrane due to their low molecular weight and remove the water from cells’ interior to prevent the formation of intracellular ice. DMSO is usually used in a range of 5% to 12% (v/v) in fetal bovine serum (FBS). A standard concentration of 10% (v/v) is used for mesenchymal stem cell preservation, allowing the maintenance of good cell viability and differentiation potential (17–19). Prior to transplantation, most cryopreserved cell products are thawed in a 37°C water bath and infused immediately into the patient. Infusion of cryopreserved products has been associated with secondary effects such as nausea, vomiting, and abdominal cramps in up to 50% of patients (20), while cardiovascular, respiratory, and neurological problems are reported less commonly (21–22). This is related with the potential detrimental effects of cryoprotectants. Cryoprotectants can be harmful to cells by causing toxicity and osmotic stress during their addition to and removal from cells. Cell toxicity is a complex topic since each cryoprotectant interacts in a different way with different cells (23, 24). These differences arise due to biochemical differences between cells but also due to different experimental conditions, such as temperature, cryoprotectant concentration, and exposure time. High concentrations of cryoprotectant are considered to be toxic to cells especially under high temperatures, where the risk of osmotic shock is greater (25, 26). Osmotic shock occurs when the cryoprotectant crosses the biological membrane, entering at a different rate than water is getting out. The rate at which a permeating cryoprotectant diffuses into the cells varies depending on the cryoprotectant, temperature, and concentration. The higher the concentration and temperature, the faster cryoprotectants tend to penetrate. In such a case, if the cryoprotectant enters faster than water, the cell cytoplasm will swell until disruption of membrane occurs (27).

However, if the cryoprotectant enters the cell slower than water exits the osmotic force becomes higher in the extracellular space, causing damage to the cell by over shrinking it. If everything is perfectly balanced, the addition of a permeating cryoprotectant causes cell initial dehydration due to osmotic efflux of water, followed by rehydration due to influx of the cryoprotectant and water. During removal of a cryoprotectant, the cells at first swell due to the osmotic influx of water and then slowly return to initial isotonic volume as the cryoprotectant and water leave the cell. However, repeated volumetric changes due to multistep washing to remove the toxic cryoprotectant can result in significant loss of functional integrity and even cell death (28). In an attempt to minimize volumetric changes, nonpermeating cryoprotectants are often added to dilution media to prevent excessive osmotic swelling during post-thaw cryoprotectant removal. Nonpermeating cryoprotectants are mainly used in combination with permeating cryoprotectants or in vitrification protocols as they promote a faster dehydration of cells (29), before direct exposure to liquid nitrogen. Sugars such as sucrose and trehalose are unable to penetrate cell membrane, so they protect it by forming a viscous shell on the surface of cells, thus regulating intra and extracellular osmotic pressure (29). While less studied, it is thought that high molecular weight polymers protect cells by the same mechanism (29). As they do not penetrate cell membrane, they present lower toxicity. Although they provide some benefits in the extracellular space, they do not provide the primary ice protection inside cells as permeating cryoprotectants do. They are therefore less effective than say DMSO used alone but combinations of permeating and nonpermeating cryoprotectants present good results in preserving, for instance, keratinocytes and keratinocyte sheets (30) (Fig. 4B). Another possibility is to force nonpermeating cryoprotectants to enter inside the cell before cryogenic preservation. Considering this, there were attempts at delivering intracellularly nonpermeating cryoprotectants through the use of electroporation (31, 32) or nanoparticles (33, 34). During electroporation, the permeability of the cell membrane is transiently increased, which allows trehalose to be loaded into cells. Loading trehalose is effective but does not significantly improve cell viability when in comparison with incubation with trehalose without electroporation (31). Electroporation is considered very harsh to cells. Besides activating some stress responses such as heat shock proteins (31), it also irreversibly damages the cellular membrane (32), compromising cell viability. The use of nanoparticles is less invasive than electroporation. Through endocytosis, nanoparticles reach the cytosol effectively without the need of rupturing cell membrane. Presenting a comparable effect on cell viability to the use of DMSO (33, 34), nanoparticle delivery of cryoprotectants can be an important alternative to the conventional approach of cryopreserving mammalian cells using toxic cell penetrating cryoprotectants.

#### Slow freezing

**Fig. 2.**
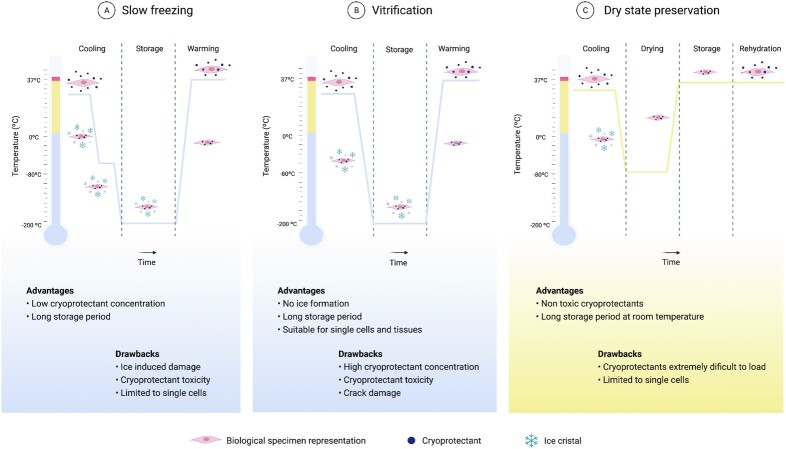
Properties, advantages, and drawbacks of long-term preservation strategies. (A) Schematic of slow freezing methodology. As low concentrations of cryoprotectant are loaded, cells dehydrate, and temperature decreases at a slow rate. Cells can be stored for long periods of time, and then rewarmed using high warming rates. (B) Schematic of vitrification methodology. High concentrations of cryoprotectant are loaded, and biological specimens are vitrified using very fast cooling rates. Storage time and rewarming is similar to slow freezing. (C) Schematic of dry state preservation methodology. As cells are coated with cryoprotectant, temperature decreases at a fast-freezing rate. After the drying process is complete, cells can be stored indefinitely at room temperatures and then rehydrated. Created with BioRender.com.

**Fig 3.**
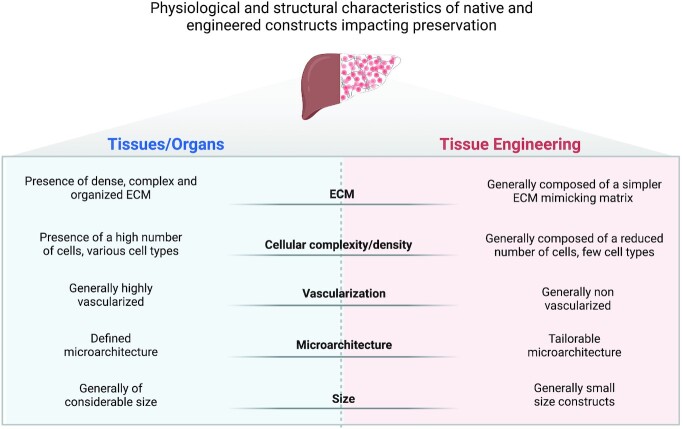
Physiological and structural characteristics of native and engineered constructs impacting preservation. Tissues and organs are defined, complex, and dense structures and therefore temperature and preservative diffusion are challenging aspects to achieve a successful preservation. The bigger and denser the structure, the more difficult is temperature diffusion. The outside surface decreases in temperature more rapidly than the inside. The outside of the tissue is forced to shrink less and the inside to shrink more, and stress caused by differential shrinkage can produce fractures. The presence of vasculature may benefit the circulation of chosen preservative; however, once more, due to its considerable size, long exposure times are needed for it to reach the whole structure of tissues and organs. Tissue engineered products are simpler, small-sized constructs, that can be tailored according to specific requirements that aid preservation. Their small size facilitates cryoprotectant and temperature equilibration, and for more complex constructs, a prevascular network can be created to improve cryoprotectant perfusion. Low cell complexity also helps in the definition of a simplified preservation protocol since a narrower set of requirements needs to be catered. Created with BioRender.com

**Fig 4.**
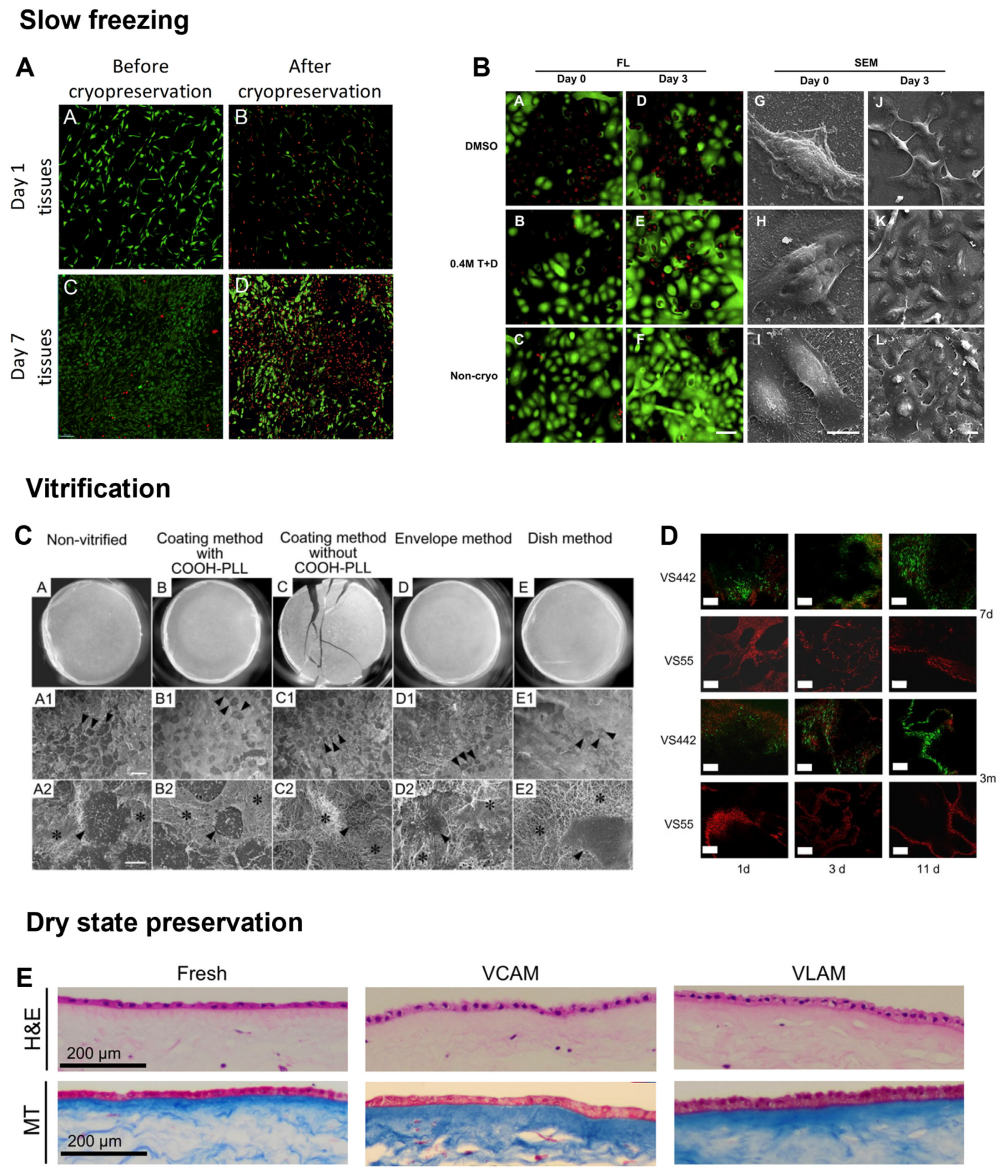
Long-term preservation strategies. (A) Cryopreservation by slow freezing of 3D human mesenchymal stromal cell tissue grafts using 10% DMSO resulted in an increased cell death after cryopreservation, especially in more mature constructs, where cryoprotectant diffusion was difficulted by matrix deposition. Adapted with permission (36). Copyright 2017, Elsevier. (B) The use of impermeant cryoprotectors such as sugars, can improve constructs viability. The use of trehalose improved tissue-engineered epithelial sheets after 1 month cryopreservation when comparing with gold standard preservation solution 10% DMSO. Adapted with permission (30). Copyright 2011, Elsevier. (C) Chondrocyte cell sheets were vitrified using different vitrification methods. These methods varied in the volume of cryoprotectant used, but in all of them, cell sheets were frozen using liquid nitrogen vapor, then rewarmed in a rewarming solution, and finally placed in a dilution solution for cryoprotectant removal. Results showed a direct link between vitrification solution volume and contact with liquid nitrogen and cell sheet damage. The use of small volumes of vitrification solution and stabilization of the vitreous state with the addition of penetrating and nonpenetrating cryoprotectants resulted in a significantly higher cell viability. Cooling samples in liquid nitrogen vapor, instead of direct immersion, prevented crack formation that occurs due to mechanical stress by liquid nitrogen boiling at the time of immersion. Adapted with permission (70). Copyright 2013, Springer Nature. (D) The development of a less toxic vitrification solution (VS442) significantly improved cell viability of tissue-engineered bone construct when comparing to commonly used cryoprotective solutions for vitrification (VS55), demonstrating that is important to develop tissue specific preservation strategies. Adapted with permission (67). Copyright 2009, Elsevier. (E) Preservation of amniotic membrane by dry state preservation (VLAM) maintained structural and functional properties equivalent to fresh state. Adapted with permission (75). Copyright 2018, *PLoS One*.

Slow freezing is the most traditional form of cryogenic preservation and has mainly been used to preserve single cell suspensions. As the name implies, cells are cooled in a controlled way through the application of a low cooling rate (Fig. 2A). Water is replaced within the cytoplasm with a cryoprotectant which reduces cell damage, and the cooling rate is adjusted in accordance with cell’s membrane permeability. In fact, the cooling rate used is very important. A too fast cooling rate will not allow cells to dehydrate, and intracellular ice will form from the remaining intracellular water. By contrast, with a too slow cooling rate, cells will suffer injury due to dehydration from the long exposure to hypertonic solutions (27), disrupting the biochemical and physical conditions required for cell survival. Usually, a −1°C per minute cooling rate is used to freeze various mammalian cell types that then are stored in vapor or liquid nitrogen (−160 to −196°C). This cooling rate allows the use of low amounts of cryoprotectant, where DMSO is the most used (35). This preservation method is very effective for cell suspensions because cryoprotectant diffusion occurs easily and cells are protected from ice formation. Slow freezing of more complex systems such as tissues and organs is more challenging (Fig. 3). It is necessary to ensure that the cryoprotectant can reach, equilibrate, and be removed from all parts of the system (36) (Fig. 4A). Furthermore, it is necessary to guarantee a uniform cooling of the structure. The latter is a pressing point of the process. The outside surface decreases in temperature more rapidly than the inside. The outside of the tissue is forced to shrink less and the inside to shrink more, and stress caused by differential shrinkage can produce fractures in the tissue (37). This is shown in several studies regarding cryogenic preservation of heart valves (38–40). The rapid deterioration observed in some implanted heart valves is thought to be due to damage of ECM fibers, mainly collagen and elastin, that occurs during cryopreservation, resulting in an accelerated valve degeneration and calcification upon implantation (38). In the case of corneal tissue, the main issue is endothelium damage since corneal epithelial cells showed no cryo-induced damage (41). In agreement with this, cell survival in cryopreserved corneal epithelial cell sheets was significant with, however, disruption of the original layered structure (42). In what respects adipose tissue, the existing reports are divergent.

Some researchers claim that there is no difference in cell viability between cryopreserved and fresh adipose tissue samples (43), whereas others have reported negative results such as low recovery of human adipose stromal/stem cells after cryopreservation (44). Necrosis of cryopreserved adipose tissue grafted into animal models (45) was also observed. An in-depth characterization of the adipose tissue implants revealed that cryopreserved tissue presented oil droplets surrounded by macrophages, abundant fibrosis, and few viable adipocytes, whereas fresh adipose tissue mostly consisted of healthy adipocytes after implantation (46). The presence of oil droplets presents a risk of clinical complications such as oil cyst formation, chronic inflammation, infection, calcification, and capsular contractures (47). However, despite cell viability loss, there is no doubt that cryopreserved adipose tissue can still be used for further human adipose stromal/stem cells isolation due to the sheer initial cell numbers.

It is evident that slow freezing has been extremely effective in the preservation of isolated cells, and it is still studied today mainly in the reproduction field. However, successes in the preservation of far more complex systems such as tissues are not so clear. This is demonstrated by the few recent publications in this regard. With increasing knowledge on the limiting factors for the preservation of tissues and organs, there is an increasing focus on the use of vitrification to that effect.

#### Vitrification

Vitrification is a vitreous, ice-free solidification of water-based solutions at subzero temperatures. Solutions pass directly from the aqueous phase to a glass state after direct exposure to liquid nitrogen, which avoids the cryoinjury induced by intracellular ice formation during the freezing process (48) (Fig. 3B). It has been used for years in the reproduction field for the preservation of oocytes (49) and ovarian tissue (50). More recently, with the growing interest in TERM strategies, this methodology has also been applied for mesenchymal stem cells preservation (51, 52). Unlike slow freezing, vitrification does not require controlled cooling and warming at optimum rates. Crystallization is avoided by using fast cooling/warming rates and high concentrations of cryoprotectants (53). Two main approaches were developed within the general methodology. In equilibrium approaches, tissue is exposed to increasing concentrations of cryoprotectant during cooling so that the system remains above the equilibrium freezing point (54, 55). In nonequilibrium approaches, the initial concentration of cryoprotectant in the system is not sufficient to prevent ice nucleation and growth, so rapid cooling rates are used, with or without the application of increased pressures (56, 57). Depending on the application, it is necessary to find a balance between cooling rates and cryoprotectant toxicity. Higher freezing rates are preferable, as it is possible to decrease the amount and time of cryoprotectant exposure, which reduces the risk of osmotic shock and toxicity. These high freezing can be achieved using carriers. Different types of carriers were developed in order to obtain different freezing rates. Conventional plastic straws have been used for the vitrification of many cells, including oocytes (58). However, in this case, the cooling rates used are not enough to lower cryoprotectant concentrations to levels that are not toxic to cells. Other carriers, such as cryo‐top, cryo‐tip, and quartz microcapillaries, have shown a significant advantage in increasing cooling rates, reducing the need for high levels of cryoprotectant (59). In addition, closed vitrification carriers, such as quartz microcapillaries and cryo‐top, are more advantageous than conventional open ones. There is no risk of viral, bacterial, and fungal contaminations that can occur when an open carrier is used, since the sample is not in direct contact with liquid nitrogen, which can be a source of contamination. Droplet vitrification is another option to achieve high freezing rates. It generates cell-encapsulating cryoprotectant droplets, followed by direct injection into liquid nitrogen (60). Unfortunately, the use of carriers or droplets is not compatible with tissue and organ vitrification due to their large volume. In fact, there are other constraints associated to their large volume and complexity. Cryoprotectant diffusion is one example. Under static conditions, transport of the cryoprotectant occurs via diffusion. It is estimated that to equilibrate ovarian tissue in the desired concentration 30 min are required at 4°C (61). Depending on the volume of the sample, this may mean even longer exposure periods to high concentrations of cryoprotectant and inability to efficiently reach the whole sample. Perfusion systems can be used to avoid these drawbacks. With these systems, it is possible to perfuse the cryoprotectant, allowing a faster equilibration period, homogeneous concentration, and smaller exposure times to toxic solutes. This approach was already tried in the past for the cryogenic preservation of rabbit kidneys, revealing promising results (62). Although not all kidneys were able to regain function, those that at least partially regained it, showed appreciable immediate function, which is not common. This because, human kidney recipients commonly require three or more dialysis treatments prior to resumption of adequate renal function. Furthermore, the impairments in the functional kidneys were comparable to those induced by ischemic stress that is known to be consistent with subsequent renal recovery. Thawing is also a concern. Successful thawing requires fast rates. For small volumes, this can be achieved by convective warming, which is not efficient for bigger volumes. For the latter, inductive warming can be applied. In this case, magnetic nanoparticles have been proposed to achieve a faster warming, reducing thermal mechanical stress and cracks, as well as devitrification (63). Both of these concerns are the reasons that may explain why the example discussed above of the rabbit kidneys (62) remains the only report of successful vitrification of large solid organs. Successful reports have been limited to small structures that can easily be cooled rapidly such as, chondrocyte cell sheets (64, 65), vascular grafts (66), bone tissue engineered construct (67) (Fig. 4D), and ovarian tissue (68). A comparison between vitrification and slow freezing of vascular grafts showed that tissue architecture is distorted by ice during slow freezing and that this was avoided in the vitreous preservation protocol (69). In the case of chondrocyte cell sheets, different vitrification methods were tested (70) (Fig. 4C). These methods varied in the volume of cryoprotectant used, but in all of them, cell sheets were frozen using liquid nitrogen vapor, then rewarmed in a rewarming solution, and finally placed in a dilution solution for cryoprotectant removal. Results showed a direct link between vitrification solution volume and contact with liquid nitrogen and cell sheet damage. The use of small volumes of vitrification solution and stabilization of the vitreous state with the addition of penetrating and nonpenetrating cryoprotectants resulted in a significantly higher cell viability. Cooling samples in liquid nitrogen vapor, instead of direct immersion, prevented crack formation that occurs due to mechanical stress by liquid nitrogen boiling at the time of immersion (70).

As some of the limitations of vitrification are being mitigated, there are at least two that are difficult to overcome. The main limitation is related with the fact that it is a very time consuming and costly process. The equilibrium approach requires lengthy periods of exposure to toxic solutes, and, at the end, it may not be possible to achieve adequate exchange of tissue water with cryoprotectants at subzero temperatures, either on a practical time scale or without exceeding the tolerable limits of solute toxicity in the tissue. In the nonequilibrium approach, the exposure of high concentrations of cryoprotectants is also an issue. Reducing the concentration of cryoprotectant would decrease toxicity but would also lead to devitrification during thawing (71). Furthermore, it is necessary to expose cells to different solutions and cooling steps before vitrification, and several washing steps to remove the cryoprotectant upon thawing (69, 70, 72, 73).

### Dry state preservation

Dry state preservation makes use of drying techniques, including freeze drying (74, 75), convective drying (76), spin drying (77), and microwave assisted drying (78), to store cells at room temperatures. In this way, samples can be stabilized in the dehydrated state for storage at room temperature, and therefore, exclude the need for freezers or toxic cryoprotectants (Fig. 3C). This concept is based in anhydrobiotic organisms, such as some tardigrades, that are capable to desiccate when exposed to extreme dry conditions because of their ability to synthesize large quantities of trehalose (79). The mechanism by which this sugar mediates desiccation tolerance is not fully understood. Trehalose is thought to depress the phase transition temperature of membranes so that they remain in the liquid-crystal state even when dry (80), inhibit protein denaturation by exclusion of water from the protein surface when cells are in the hydrated state (81), inhibit protein aggregation during heat stress (82), and preserve the structure of proteins in the dry state (83). Furthermore, trehalose has a high glass transition temperature and causes the formation of stable glasses during drying (84). Freeze-drying has been applied for the preservation of proteins and liposomes for pharmaceutical applications, using trehalose as the main protectant (85). Freeze-drying of more complex systems, like mammalian cells, continues to be a challenge. One of the main problems is the impermeability of cells to trehalose since most tissues of interest lack a trehalose transporter. Over the years, several protocols to deliver trehalose intracellularly were tested. One of the first protocols attempting to load cells with trehalose was through phospholipid-phase transition via thermal, osmotic, or electric stress (86, 87). Although effective, allowing trehalose to cross the plasma membrane and accumulate inside cells, some collateral damages are observed, mainly membrane stability loss. Other approaches consisted in the use of thermally responsive pluronic nanocapsules (88) and direct microinjection in large cells such as oocytes (89), although the latter was revealed to be inefficient for smaller cells. A different approach involved genetically engineering cells to make their own trehalose, namely human fibroblasts expressing *Escherichia coli* genes using an adenovirus vector (90). These techniques require direct manipulation of the cells, exposing them to nonphysiological conditions. Membrane modification or insertion of carriers or genes are difficult to implement due to regulatory concerns. A method that does not require any of these manipulations is based on fluid phase endocytosis. The introduction of trehalose into the intracellular space occurs through a mechanism where cells internalize their own plasma membrane and macromolecules from the external environment. It is a time, temperature, and energy-dependent process where the internalized molecule does not bind to the cell surface contrary to what happens in receptor-mediated endocytosis. This cellular process has been used to load trehalose into mammalian cells, by simply incubating them in the presence of a high extracellular concentration of trehalose. When applied to human blood platelets, it allows their storage and survival for 2 y at room temperature in the desiccated state (91). Using the same technique, it was possible to load this sugar intracellularly in mesenchymal stem cells (MSCs), confirming that the uptake was temperature, incubation time, and extracellular trehalose concentration-dependent (92). Similar experiments were also reported in other cell types such as mouse J774 macrophage cells (93) and mouse 3T3 fibroblasts (94), as well as in the preservation of tissues (75). Freeze-dried human amniotic membrane maintained structural and functional properties equivalent to the fresh ones (Fig. 4E). As mentioned before, fluid phase endocytosis does not happen through the specific binding of membrane receptors, but by the cell internalizing part of its environment within a vesicle of the plasma membrane. Thus, it is a simple approach that reveals itself to be efficient and at the same time less invasive to the cell, although requiring long incubation times for still very low intracellular trehalose concentrations.

## Short-term preservation

### Hypothermic preservation

**Fig. 5.**
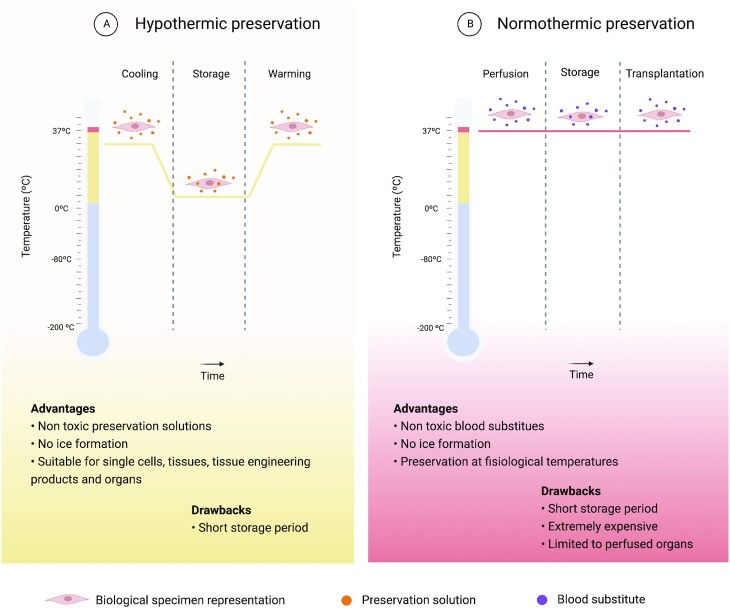
Properties, advantages, and drawbacks of short-term preservation strategies. (A) Schematic of hypothermic preservation methodology. Preservation solution is added to the biological specimen, which is stored at hypothermic temperatures for short periods of time, until rewarming. (B) Schematic of normothermic preservation methodology. Blood substitute is perfused into the organ in a humidified organ chamber and maintained in physiological-like environment until its transplanted. Created with BioRender.com.

Although cryogenic preservation is considered the gold standard for single cell preservation, with significant benefits accomplished for cell-based therapies, the challenge for preserving a tissue or tissue-engineered construct, while keeping cell viability and the structural properties of the supportive matrix, remains a challenge. Therefore, few studies show its use in complex systems such as organs, tissues or tissue-engineered constructs. Due to the great advances made in the TERM field, new preservation methods that ensure the preservation of cell function in complex systems are needed. Short-term preservation using hypothermic temperatures can be optimized to a preservation time frame that allows time for transportation, while ensuring quality, safety, and efficacy testing before administration to the patient. The definition of hypothermia changes depending on the field it is applied. In the emergency medicine context, 4° of hypothermia are defined that do not exist in the preservation context. Furthermore, inside the preservation context, there is no consensus in the terminology used, as some authors classify temperatures around 20°C as subnormothermic or even room temperature. Considering this, and for the purpose of this article, all temperatures below 35°C will be considered as hypothermic temperatures. The use of hypothermic temperatures for preservation is widely inspired by hibernating animals and their ability to decrease body temperature. By decreasing body temperature, they are able to decrease cells metabolic activity and, in turn, their energy needs. Hypothermia has been used for a long time in several medical applications. This includes the reduction of the heart’s temperature during cardiac surgery, to reduce the risk of myocardial ischemia (95) and to limit the severity of traumatic brain injuries (96). Such temperatures slow energy dependent processes like protein synthesis, transport systems, and cell cycle progression. This way, cells can be suspended during a short period of time with a simplified method that avoids cell and ECM damage from ice formation and changes in solute concentration caused by extreme temperature shifts during freezing (Fig. 5A). However, exposure to hypothermic temperatures does have some damaging effects. When the metabolic activity of cells is slowed down, there is a mismatch between ATP supply and ATP demand pathways. In consequence, ion transport systems such as Na^+^/K^+^-ATPase are inhibited and the normal balance between Na^+^ influx and K^+^ efflux is disturbed, in favor of Na^+^ influx. This leads to an accumulation of Na^+^ that is exacerbated by hypothermia induced activation of the Na^+^/H^+^ exchanger, leading to cell swelling (97, 98). This mismatch is also responsible for the increase in reactive oxygen species production, especially in the mitochondria, and for the suppression of the enzymatic processes responsible to decrease their concentration (97, 98). Although there is no consensus, growing evidence suggests that the production of reactive oxygen species not only occurs during hypothermic storage, but continues during warming (99–101). Considering this, effective hypothermic preservation must use preservation solutions that provide optimal concentrations of ions and impermeant molecules to maintain ionic and osmotic balance, prevent the formation of free radicals and supply energy substitutes. A variety of preservation solutions were developed taking these factors into consideration. The first preservation solutions were developed for organ transportation and are used until today for that purpose. In fact, static preservation at 4°C is, with the help of these solutions, the gold standard to preserve organs during transportation from the donor to the recipient (102). More recently, with the growth of TERM, some of these solutions have been tested or modified and new ones have been developed with TERM products in mind. University of Wisconsin solution, histidine–tryptophan–ketoglutarate solution and, HypoThermosol (HTS) are the most used ones and have been tested in a large diversity of cells and tissues including human pluripotent stem cell-derived cardiomyocytes (100), bone marrow mesenchymal stem cells (103), endothelial cells (104), renal cells (105, 106), and hepatocytes (107–109).

**Fig 6.**
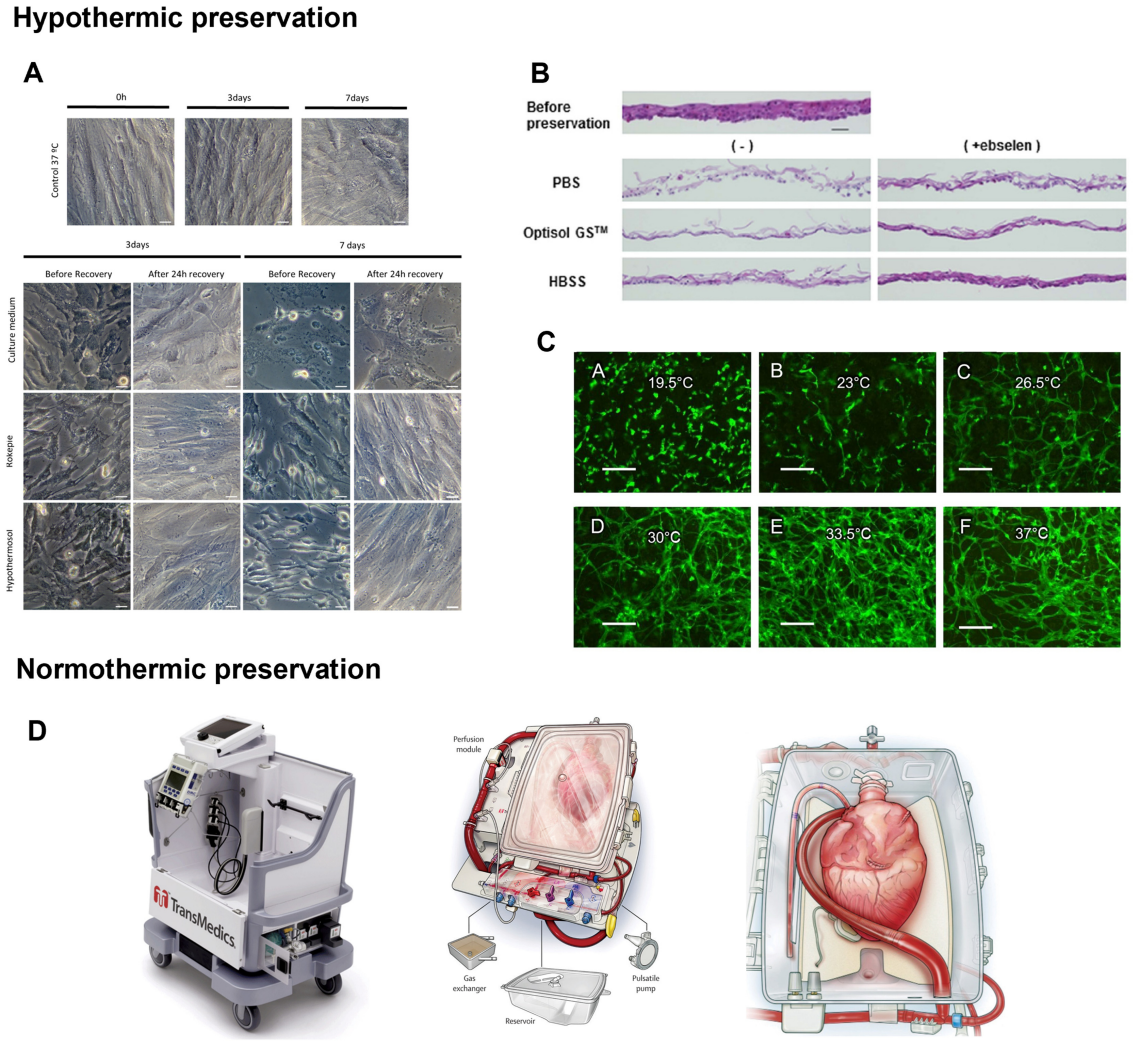
Short-term preservation strategies. (A) Hypothermic preservation at 4°C of cell sheet-like confluent hASC cultures using Rokepie and Hypothermosol as preservation solutions, retain typical spindle-like shape after 24°C recovery period, when comparing with unsupplemented condition. Adapted with permission (115). Copyright 2019, *PLoS One*. (B) Similarly, the use of other preservation additive, ebselen, improved hypothermic preservation. In both HBSS and HBSS+ ebselen, the morphology and stratification of the cell sheets were well maintained, and the staining intensity of eosin was improved by ebselen supplementation. Adapted with permission (114). Copyright 2016, Springer Nature. (C) The use of milder hypothermic temperatures can also be beneficial. Exposure to hypothermic temperatures of 33°C developed denser prevascular networks than at lower hypothermic temperatures or even at physiological temperatures. Adapted with permission (135). Copyright 2018, Elsevier. (D) A clinical study, where the clinical outcome of patients receiving a heart transplant preserved by normothermic ex-vivo allograft blood perfusion using the Organ Care System by Transmedics, USA, was compared to that of patients receiving a heart transplant preserved by static cold storage. When comparing hearts preserved with the Organ Care System or standard cold storage, it was seen that both strategies lead to similar short-term clinical outcomes. Nevertheless, the normothermic strategy was able to prolong the preservation time from the standard 6 to 9 h without increasing immediate organ damage. Adapted with permission (141). Copyright 2019, Elsevier.

HTS has been particularly successful for the storage of cells at 4°C (110). This preservation solution has shown how important is the formation of reactive oxygen species and the role they play in cell death. The first formulation of this solution did not include any potent antioxidant, which was reflected in poor cell survival (110). The addition of Trolox changed this outcome dramatically (105, 110). But, in fact, the design of a successful preservation solution should comprise not only agents to regulate cellular levels of free radicals but also of electrolytes such as Na^+^, K^+^, Ca^+^, Mg^+^, and Cl^−^ to counteract ionic imbalances, at least one simple sugar such as glucose to serve as a source of energy, a biological pH buffer effective under hypothermic conditions such as HEPES and an effective impermeant such as sucrose or mannitol to counteract cell swelling during cold exposure. In the specific case of organ preservation, the presence of a macromolecular oncotic agent having a sufficiently large size to limit its escape from the circulatory system and being effective to maintain oncotic pressure equivalent to that of blood plasma, is also crucial (111). The addition of other compounds such as inhibitors of the apoptotic cascade, like FK041, or of the Rho-associated kinase, and modulators of cellular interaction with the ECM, such as RGD, may improve the antiapoptotic effects of these preservation solutions and prolong the duration of preservation (105, 110, 112). However, the addition of inhibiting drugs will require regulatory justification to ensure there will be no adverse effects in the patients. This does not invalidate that other supplements can be used to improve preservation at hypothermic temperatures, and key potential components are potent free-radical scavengers, or molecules that activate reactive oxygen species stress response pathways. Ebselen and SUL-109 are examples of this type of molecules that were already tested for hypothermic preservation. Ebselen is a seleno-organic compound with antioxidant activity that mimics glutathione peroxidase action (113). It catalyzes the reduction of reactive oxygen species, thus reducing cell damage during preservation as it was observed in the preservation of oral mucosal epithelial cell sheets (114) (Fig. 6B). SUL-109, a 6-chomanol derivative, protects cells by preserving the mitochondrial network structure and activating mitochondrial complexes I and IV, therefore maintaining ATP production and preventing ROS formation. It’s effectiveness was demonstrated in the preservation of human adipose stromal/stem cells without reducing their multilineage differentiation capacity (115, 116). These compounds can be added directly to cell culture media and this mixture used as the preservation solution. However, given that typical culture media only provides pH buffering for culture-specific CO_2_ concentrations, care has to be taken to ensure proper CO_2_ concentrations at hypothermic temperatures, either through special incubators or proper sealing of the cell containers. Another option is the use of media able to provide pH buffering at ambient CO_2_ levels, such as Hank’s Balanced Salt Solution or Leibovitz’s L-15 medium (117, 118). Other different approaches rely on the use of cellular encapsulation to maintain membrane stabilization and subsequent protection against the osmotic shock and mechanical stress during storage and recovery. Alginate encapsulation was demonstrated to be effective in the preservation of human bone marrow stem cells, mouse embryonic stem cells, and human limbal epithelial stem cells at room temperatures (119, 120), and rat hepatocytes and recombinant baby hamster kidney cells at 4°C (121, 122). The use of hypothermic temperatures, in combination with alginate encapsulation, was shown to protect cells against induction of apoptosis not only when compared to cells preserved at room temperature, but also when compared to freely suspended hepatocytes (123). Even though cell encapsulation offers structural support to cells and protection from cold induced injury, cells still require retrieval and processing once delivered to the medical site, causing mechanical stress in cells. The preservation of more complex tissue engineering structures also benefits from the absence of ice formation that hypothermic temperatures provide. A comparison between cryogenic and hypothermic preservation of tissue engineered bone grafts demonstrated structural alteration of the extracellular matrix as well as cell death due to ice formation, which was not observed at 4°C (124). In the case of cell sheets preservation, a direct comparison between these two preservation strategies has not been made although preliminary results in our lab indicate extensive ECM damage brought by ice formation. On the other hand, the preservation of human adipose stromal/stem cells and oral mucosal epithelial cell sheets at 4°C did not significantly compromise cells viability, function, and ECM structures (114, 115) (Fig. 6A). For other tissue-engineered products, the use of milder hypothermic temperatures, around 20°C are more indicated. Such is the case of some approved cell therapy products, including Epicel (125) and Apligraf (126). The effectiveness of using milder temperatures for single cell preservation of some cell types was also verified. Some reports directly comparing different hypothermic temperatures show that milder temperatures were, in fact, better (127–129). Fourty-eight hours of storage of placental MSC under 20°C ensured the preservation of cell number and metabolic activity whereas preservation at 4°C demonstrated to reduce cell number only after 24 h (127). In the case of a recombinant chinese hamster ovary cell line, three different temperatures were compared. After preservation at 4, 12, and 24°C no differences were observed after 3 days of storage in bioreactors. However, after rewarming at 37°C for 16 h, viability was reduced to 30% in cells preserved at 4°C (128). Conversely, other comparative studies show that lower hypothermic temperatures are more advantageous when comparing to milder ones (130–132). Which is the case of hematopoietic progenitor cells that experienced greater colony forming unit loss when stored for 72 h at 20°C compared to 4°C (132). In fact, a study involving cell lines such as WI26, MG-63, HeLa, HMEC, and HBME-1 showed that exposure to milder hypothermic temperatures affects the same mechanisms as exposure to lower hypothermic temperatures (133). For instance, after 5 days of storage at 25°C, morphological alterations were observed that were not restored after recovery at 37°C. Cell content was affected after rewarming and significant apoptosis induced during preservation remained elevated after recovery. Furthermore, ROS levels in cells kept at 25°C for 5 days were similar to the control but increased after rewarming. Still, this study only addressed the impact of milder temperatures, not making a direct comparison with preservation at 4°C. Curiously, incubation of tissue-like constructs under hypothermic temperatures has been studied out of the context of preservation. The cultivation of adipose tissue-derived microvascular fragments (ad-MVF) at 20°C, under a controlled atmosphere with 5% CO_2_ and regular culture media, increased the proliferation capacity of endothelial cells and perivascular cells, as well as their in-vivo vascularization capacity when compared to normothermically cultivated ad-MVF (134). In the case of vascularized cardiac cell sheets, cultivation at 33°C developed denser prevascular networks than at physiological temperatures (135) (Fig. 6C). This suggests that milder temperatures can be beneficial to address the critical issue of vascularization of tissue engineering constructs. Hypothermic temperatures were shown to be able to preserve with little damage different types of structures, ranging from the simplest ones such as single cells all the way to the most complex such as organs. Nevertheless, damage limitation still is inversely proportional to preservation time. This implies that hypothermic preservation can only be used for relatively short periods of time, which is the major drawback of this preservation strategy, limiting their range of applications.

### Normothermic preservation

As demonstrated above, damage brought by hypothermic preservation is proportional to preservation time. In the case of organs, even with the most effective preservation solution, cold storage leads to graft injury due to cold exposure (97) and ischemia (136). In this context, the use of normothermic temperatures with the help of perfusion has gained some attention. Organ preservation by normothermic machine perfusion is based on the hypothesis that preservation injury can be minimized by avoiding cooling and hypoxia. It consists in the delivery of oxygen and nutrients through a perfusate, and maintenance of normal body temperature, that together allow normal physiological metabolism during organ storage (Fig. 5B). The choice of perfusate is organ dependent but always with the goal of guaranteeing an adequate delivery of nutrients and oxygen essential to maintain cellular and vascular integrity. The typical circuit contains a humidified organ chamber, perfusion solution, reservoir for perfusion solution collection, oxygenator and optional leukocyte filter connected via sterile tubing. This configuration has been adapted by several companies to develop the existing commercial normothermic perfusion platforms, including Organ Care System, OrganOx metra, XVIVO Perfusion System, Organ Assist, and Vivoline LS1. These devices have been employed in a variety of preclinical and clinical studies of several organs such as liver (137–139), lung (140), heart (141), and kidney (142) and have demonstrated an ability to successfully preserve organs under normothermic machine perfusion. In the case of liver and lung transplantation, remarkable improvements have been accomplished. A recent study comparing the use of Organ Care System with cold storage for 320 patients receiving a double-lung transplant showed that 72 h post-transplantation, the incidence of primary graft dysfunction was reduced when normothermic temperatures and perfusion were used (140). In the case of liver transplant, the level of liver damage within 7 days after transplantation was 50% lower in the normothermic preservation than in the cold storage. A 50% reduction in the proportion of livers that were discarded before transplant and a 54% increase in the duration of preservation in the normothermic preservation (137) were also demonstrated. In what regards heart transplantation, the successes were not that evident. When comparing hearts preserved with the Organ Care System or standard cold storage, it was seen that both strategies lead to similar short-term clinical outcomes (141). Nevertheless, the normothermic strategy was able to prolong the preservation time from the standard 6 to 9 h without increasing immediate organ damage. It should be noted that all the studies presented in this section were not intended to study the increase in preservation time with the use of normothermic machine perfusion, but rather a comparison with the standard method, hypothermic preservation (Fig. 6D). However, considering that these are new procedures, they implied changes and the consequent acquisition of new routines that led to an increase in the preservation time compared to hypothermic preservation. The collected data gave a first indication that is possible to safely extend preservation times without compromising outcomes. Nevertheless, more focused studies are needed, as prolonged preservation times could balance the inequalities in donor availability in different regions. Unfortunately, the gains in viability and preservation time may not be worth the costs. The basic requirements to provide metabolic support in a normothermic preservation system are huge. An organ chamber, perfusion pump, tubing, an oxygenator, heat exchanger, and monitoring devices to measure flow, pressure and temperature are necessary, which represents high costs and increased logistical difficulties when considering the additional equipment needed. In what regards TERM constructs, the progresses accomplished by this technique cannot be applied in the overwhelming majority due to the lack of a mature vessel networks that enables the use of perfusion.

## Conclusions and future perspectives

Remarkable progresses towards the development of engineered tissues and organs have been accomplished in the field of TERM. With these advances, comes the requirement for effective preservation methodologies that can address logistical challenges related with widespread clinical application.

When considering long-term preservation strategies, slow freezing represents one of the oldest storage methods. Other methods such as vitrification with the use ultra-fast cooling rates, and, more recently, dry state preservation have also been explored to address the challenge of eliminating cryoinjury while mantaining a prolonged preservation time. Research to improve cryopreservation protocols, using perfusion systems to deliver cryoprotectants more uniformly, using nanoparticles to deliver nonpenetrating, and less toxic cryoprotectants inside cells, or using magnetic nanoparticles to achieve a more uniform rewarming, are some of the alternatives for application to therapeutically relevant cells, tissues or tissue-like constructs. Studies on emerging approaches are in their early stages, and abundant research gaps need to be filled to enable further improvements and more function-accessible mechanisms. Hypothermic or even normothermic preservation can facilitate short-term preservation and transport, increasing construct accessibility. In the case of hypothermic preservation, new additives have been tested, namely apoptosis inhibitors and free radical scavengers, in order to tackle the adverse effects of low temperatures. Finally, normothermic strategies, combined with perfusion systems, have revolutionized the organ preservation area.

However, there is still a long way to go. As new protocols using encapsulation are being developed, their safety for clinical use needs to be tested. Furthermore, preservation of tissue-engineered products, cells, tissues, or organs cannot be seen as “one size fits all” in the sense that it will be impossible to find a universal preservation strategy for all products. Especially in the case of tissue-engineered constructs, that sit somewhere between cells and tissues. It is necessary to understand the specific characteristics and needs of each product, and then choose the one that best suits each application. In other words, the preservation strategy needs to be tailored around the object of preservation. Therefore, while a lot has been accomplished in the field of preservation, new strategies capable of streamlining the application of TERM-based products are needed.

## Supplementary Material

pgac212_Supplemental_File

## Data Availability

There are no original data in this work.
