# Supplementary material for: Long-term and short-term preservation strategies for tissue engineering and regenerative medicine products: state of the art and emerging trends
Source: PNAS Nexus. 2022 Sep 30;1(4):pgac212. doi: 10.1093/pnasnexus/pgac212 (PMC9802477; doi:10.1093/pnasnexus/pgac212)
Supplement: pgac212_Supplemental_File [file pgac212_supplemental_file.docx]

**Table S1.** Summary of studies reporting strategies for the preservation of tissue engineering products.

| **Preservation method** | **Tissue engineering product** | **Preservation solution** | **Freezing protocol** | **Thawing protocol** | **Storage time** | **Ref. and year** |
| --- | --- | --- | --- | --- | --- | --- |
| **Slow freezing** | Corneal epithelial cell sheets | 10% (v/v) glycerol  or  10% (v/v) DMSO | -1ºC/min to -80ºC followed by liquid nitrogen | 37ºC water bath | Up to 12 weeks | [42]  2005 |
|  | Chitosan-gelatin membranes + human keratinocytes | 0.2 m trehalose 10% (v/v) DMSO 50% (v/v) FBS  or  0.4 m trehalose, 10% (v/v) DMSO, 50% (v/v) FBS  or  0.6 m trehalose, 10% (v/v) DMSO, 50% (v/v) FBS  or  10% (v/v) DMSO, 50% (v/v) FBS | 4 °C for 30 min followed by liquid nitrogen  or  4 °C for 30 min,  −20 °C for 2 h,  −80 °C overnight  followed by liquid nitrogen. | 37ºC water bath | 1 month | [30]  2011 |
|  | Collagen porous dics + human adipose stem cells | 10% (v/v) DMSO | -1ºC to -80ºC followed by liquid nitrogen | 37ºC water bath | 24h | [36]  2017 |
| **Vitrification** | Partially demineralized porcine bone matrix + Canine bone marrow derived mesenchymal stem cells | 40% (v/v) DMSO,  40% (v/v) Euro collins  or  31% (v/v) DMSO, 23% (v/v) propylene glycol, 17% (v/v) formamide, 28% (v/v) euro collins | Liquid nitrogen | 37ºC water bath | Up to 3 months | [67]  2009 |
|  | Chondrocyte cell sheets | Equilibration solution  10% (v/v) DMSO and 10% (v/v) ethylene glycol,  Vitrification solution  20% (v/v) DMSO, 20% (v/v) Ethylene glycol, 0.5 M sucrose, and 10% (w/v) carboxylated poly-l-lysine  Rewarming solution  1M sucrose | 24ºC for 20 min in equilibration solution,  4ºC for 5 min + 15 min vitrification solution,  20 min liquid nitrogen vapour | 38ºC for 90s heating plate, followed by 24ºC for 1 min in rewarming solution | 4 weeks | [65], [70]  2013  2017 |
| **Hypothermic preservation** | Rat dermal fibroblasts cell sheets | DMEM | 20ºC |  | 8h | [117]  2008 |
|  | Oral mucosa epithelial cell sheets | 10 μM or 100uM Ebselen | 4ºC |  | 7 days | [114]  2016 |
|  | Neonatal rat cardiac cell sheets | Basal media | 19.5 °C or 23 °C or  26.5 °C or 30 °C or 33.5 °C |  | 3 days | [135]  2018 |
|  | Human adipose stem cells cell sheets | Hypothermosol  or  Rokepie | 4ºC |  | Up to 7 days | [115]  2019 |
|  | Decellularized cow bone disks + human iPSC‐derived mesenchymal progenitor cells | PBS | 4ºC |  | 2 days | [124]  2020 |
|  | Epicel® | DMEM | 13ºC – 23ºC |  | 24h | [125] |
|  | Apligraft® | Agarose nutrient medium | 20°C-23°C |  | 10 days | [126] |
